# Supplementary material for: Arid3a regulates nephric tubule regeneration via evolutionarily conserved regeneration signal-response enhancers
Source: eLife. 2019 Jan 8;8:e43186. doi: 10.7554/eLife.43186 (PMC6324879; doi:10.7554/eLife.43186)
Supplement: Supplementary file 1. [file elife-43186-supp1.docx]

Supplementary File 1 - The primer sequences used in this study

| CNSs | Amplified length (bp) | Target sequences | Primer sequence |
| --- | --- | --- | --- |
| *lhx1*-CNS1 | 133 | TACAATTGAACTGAATTAATTTGCATGACAAAATTGGCTCGGGTATTCATGTGTTGCGGGAAGACAAGTGTAACGTTACAAAGAACAAGTAACCATGGGAAAGGTTAATGCAGACAATACGCAAGCACAATAC | TACAATTGAACTGAATTAATTTGCATGACAAAATTGGC |
|  |  |  | GTATTGTGCTTGCGTATTGTCTGCATTAAC |
| *lhx1-*CNS2 | 455 | CACTCATTTTCTATAGAAATTTCCCAGATTAGATTTGTAAATCAAAAACCAATATATAACATGCAAGAATTGATCTGTTTTATATTCCTCAGTTACATTTTTGTGAAGCACATTTAGAAGATTTAGAACGTATTTGCGCCTGCTGGTTGAGCCATTATATGCTTGAGTTCATACATCTATTTGTGAGGCTATAAAACAGGCATCGACTACCCATGTAAATTGTGTTCTCCACAATAAATTATATGCATACTTGCAAAAATACATAACTGCTGTTGGTTGGGTTATGTAGCTTGAATACTAATGATGTGTAAAGTTGCATTGTCCCTCTTCTTGAGCCTAATTTAGCTGCTATATCAGAAATTATGTAGGAAAAAACACTTTATCCTCTCACTGAGAAATAAAATGGCACTTACTGACACCTACATATCTTTTTTTTATGAAACACCACCATTTTCATATTCATTTTGCCTGCTCTGGGTTCATGGAAAAGTCGCTCCTGTGCCATGCCCATAAAAGAATTTAGTACATAAAAAT | CACTCATTTTCTATAGAAATTTCCCAGATTAGATTTG |
|  |  |  | TTTTTATGTACTAAATTCTTTTATGGGCATGGC |
| *lhx1*CNS3 | 368 | TATCGCATAGCTTAGTACTTTGCAAAAACACGGAACCGCAAATCCAATAAAATTGCAAATTTCACTGTATTTCACAATATCCTAAATAAACATGAACAGCATGCATTTTTCTTTCTGGCATCAAATATTAATCTGTCCGTGGTTGGAAATGCGCTTTCCATCTTGTGTGTGCTCACATTGAGTAAACACTTGGGAGATTGCTATTCCAGACCTTTCATTCTCTCCCAGAGTCAAGAGCATGACAAATTCTTCTTCTAATTACACCTGCCACTGTTTGCCTTCTAATACTGTAAATCCTCCATCACATAGTTTTTTTTCAGATTCAGGATTAGCTTCCTTTGCTGTCATACAAATGACTAAGGTACAGG | TATCGCATAGCTTAGTACTTTGCAAAAACACG |
|  |  |  | CCTGTACCTTAGTCATTTGTATGACAGCAAAG |
| *lhx1-*CNS4 | 340 | TCAGAAGTCCAGTCCAATCGCTTTTCACAGTGTCTGTACTCACGCCAGGCTATGGATTAACCAGGGAGTGATACAATAGGCACATATGCTCCAGTGTCCAGTCCTGCCTATTCATATGTGTGTTACTTAGTCTAATAAGGATTAGAGTTAATGAATTGGAAGGAGTTAACAATATAAAGAGGCAATAGAAAAAGGCCTAAGTAGTGGTAATTCCATTTCATGTAGCTTTGGTGTTGCTTTTCTTTCAGCGAATAGGTGTAATCCTCTTTGAAATCAAAGGTGGTACTAAGTATGAATTTAAATTTGACATTTACAGCGACTGTAAGATTAAGTCTTCCA | AAGTCCAGTCCAATCGCTTTTC |
|  |  |  | TGGAAGACTTAATCTTACAGTCGCTGTAAATG |
| *lhx1-*CNS5 | 323 | CAGCTAAGAATACAGTCCTCACATTGCCTCACATTGAGTATCTGTCATTCATTGAGTCGCTTTCCCCAAATGTATTTTTTTTCTTGTGGAAATAAGAAGAATTTCAAATTAGTTAACTGAAAAAAAAAAAAAACAGACAGGTCCACATGTACTTGGAGAAATGAACAAAAAAATAACTAGAACTTTGTCAAACTTTGAGTTCATGAACAATTTAAAACATGCCTAGTTCACTGAAATATGTCGATTATTTTAGGTAAAAAAATCACGTATTTTTTGACTCAGTCCATTTAATTTCAAGAACAAAGTCCAGTCGAATGTGCAC | CAGCTAAGAATACAGTCCTC |
|  |  |  | GTGCACATTCGACTGGAC |
| *lhx1*-CNS6 | 521 | ATAGTGGGAAAATTGCAGAAAACTAGCAGAAAAATTGAGCAGTTGGCTATTTATGACTCAGACACAATACCTAAATAAAATAGCCGATCTAATAACTGTTTGTGAGCAAGCTTTTCCCTGCTTCTTAGCACACAAAGATTTCCTTTATTCCTTTGTAGCTTCAGCAGCTATTTCAGAAAAGAAGAAATCAATAAACCTTTCCATCTTTCAATCTTTGCCCCAAAAGGTGTTTTTGGGTAGCGTTTTGTCATTCATAAGCTGTGGCGACAAATCCTTGACATCATTCATGGGTAATATCGATCACTTCTTCTTTTGCACCAAAGGACAAACAGAAATCATTTATCTGTCTGTACTGTGAAACAGTAAAAGACAAAAGGATCTGCCAGCCTTCCATGACAATCAGCAGCTGAAGGTATTAACAAGAGGTCAAGTTGAAAGGTGAAAGGCCAAACAGGTTCTCATCAGTTTCTCACAGCTCTCCAAGTTGGGAAGAAAACAGCTGTCTCCCATTAGCTCAATCC | ATAGTGGGAAAATTGCAGAAAACTAGCAG |
|  |  |  | AAACAGCTGTCTCCCATTAGCTCAATCC |
| *lhx1*-CNS7 | 304 | AGACTAGATGAGAGATTGAAAAAATGAGAGCCTCTTTGGCACAGGAATGCTGCTGCTATTGAAAGTGAGTAAATTTTAATGdAAACGGTGCAAAAATGACATCATTTACTCAGAAATCATTAATTTGTCAGCATTAATATTTCAGCTACAACATTGTCTGTCATTTTATGCCTGTGATGACTGATATATCTATAAAAGTATGCTGCTAATTTACTGAATATTTATGTGCAGTGTCATTTGAAGAGGACTGGAATTTATTTCCCTATTCAGTCAGCTCAGAACATTCCTAAAATGGAAGACTATA | AGACTAGATGAGAGATTGAAAAAATGAGAGCCTCTTTG |
|  |  |  | TATAGTCTTCCATTTTAGGAATGTTCTGAGCTGAC |
| *lhx1*-CNS8 | 199 | CTGTTTCTCTTCTCCACCCACCACATTAGCCTCTGAATACTCTCATTATACCATTACTAATTAGAAATTCTCTCCAATTGGCGGCTGCATTAAAAAAAGACAGTGTGCCGAGCATATGGCATAATATCCTATTAATGAACACTCTAAATTCAAACTCAGGTCAGCTGTTCATTTCTTGTTCATGTCACCCAGGCAAGAA | CTGTTTCTCTTCTCCACCCACCACATTA |
|  |  |  | TTCTTGCCTGGGTGACATGAACAAG |
| *lhx1*-CNS9 | 175 | ATGTAGATCTCTGTGACCCCATTACATGTTTTTCTAACCATAAACTATCTGATTTATATTCATTAACCAGTACTAGAAAGCAGCACTGGCATATGTTTAGTATGGAACATGCCTTAGCAGAAGGGTGGACAAAATTTCAGTTAATTAAGTTGTTGTAATGGCAGCATAGTACAAA | ATGTAGATCTCTGTGACCCCATTACATGT |
|  |  |  | TTTGTACTATGCTGCCATTACAACAACTTAATTAAC |
| *lhx1*-CNS10 | 366 | CAGCATGATTTCTCCAGCAGATATTCGGCAATTCACTACAGACAGTAAACAACTCTTCTTTTTGGAACTATGCAGGGGAACACAGTAGGGATGCTGGAAAAGCAGGGGTTAATAGAAAGTAAAATGTTGTTTTTATTGACAAAACTATTTGCACACATAAAGCTAAAATTTAGTTTTATTGTTGAGTACATCATAACGTATCAGTATCAACAGTATCACAGCATATGAACAAAAAAGTTATATCTCATTATTTGCTGTTTATTATTTACAAGTAAACCACCTTTTCTATTAATATAACTCACCTTTCTATCAAGGCAATTATTCTCACAAAACAAGAACATAACATATAACAAAGTGCAGTGAGCC | CAGCATGATTTCTCCAGCA |
|  |  |  | GGCTCACTGCACTTTGTTA |
| *lhx1*-CNS11 | 371 | CTGCACATCTGATCAACAAATTGCTGATTTAATTTCAATTATTGCCCTTTAATAAGGCATCTTAGCATTTCAATTTTTTTATCCATCTCTCTTGTTTTAATCCCTTTAGCAGTGAAACATTTTTGGCAATTTTTGGTTTCTTTTTTTTCTAAACTCCTCTTATCTGTGCACATTATAAGACTAATCCTGAAGTCCTAGTCAGAACATTCTTTATTCTGTAAAGAAGTGGGTAGTAATCTGAATCAAAGAAGGTAGTTCCTGTGATGGTTTTAATAATAACTTTGATAACATTCATAATACTTTTTTACCCTTCTTTTTACTGTAAAAAGTAGGGATACGCGTAGATAAATGAAAGAGTAGCATTTGCAGCC | CTGCACATCTGATCAACAAA |
|  |  |  | GGCTGCAAATGCTACTCTT |
| *lhx1*-CNS12 | 482 | GCCTGATCAGTTTAGAAACGTTGCATATGTTTCACTAAACAGATGGGTCTTTAGGGAGCGTTTGAAAGTCTGGAAGGAAGGGGAGGCAGAGAATTCCAGAGACAGGCAGAAGCCCGGGAGAAGTCTTGCAGTCGGAAAGGGGATGAGTTGATGAGAGGAGAATAGAGGTGAAGGTCAGAGGCAGAATGTAGGTTGTGTGAAGGAGTGTATTAGTAAGGGATTTCTATGTAAGTGTTAGTTATTTGAATTTGATTCTAGAGAAGATTGAAAGCCAGTGAAGAGATATGCATAGAGGAGCGACAGATGTGGATCGACGAGATAGATGAATGAGTCTGGCAGCAGCATTTAGAACATATTGTAGTTGGGAAAGGTGATGTGTTGGGATGCCTGTAAGAGGTAGGTTACAGTAATCAATGCGGGAAATGATCAGAGATTGGATGAGTTTTATTTGTGTCTAAACTGAGGTAAGGGTGTATTCGGGC | GCCTGATCAGTTTAGAAACG |
|  |  |  | GCCCGAATACACCCTTAC |
| *lhx1*-CNS13 | 492 | CTTGAAATGGGGAGGAGAGAAAGAACTTAAAATTCTCCACAAGCAGTCAAGCGGAACCTATTTTCTCTTACAATGCAATTAACAGCGCCAACATTTACAAAATATGATTACAAGCACTTTGGTTAAGTAGAGAAAACTATATTAGATAGCAGCGAGGGGTTGATCGTTATGTGCATTTTTTTCTTTGCAGAGAGAGAGACTTGCATTTTCTTTGTGCAAATAAAGAGGTCCTAAATTCTCCATAGGCACTAAAATCAATGCAGTGGAACCGTGATAACAAACACAGGCTCCACTTTTTGCTACATCCCCCAATTAATTAAGCCTTTCACGGGCATGTAATTAGGAACATTAGCAAGTCATAAAATTGCAGTGAGTCTCTAAATTATGTGCTGGCCCTCAGCAATTTGTTTTAAATTCCTTTTGAAGACAATGGCACTGCGGGGCCCCATAGATCTTCATACAAGCCTACTGTTTACTTCTTTATGACTCTAC | CTTGAAATGGGGAGGAGAGAAAGAACTTAAAATTC |
|  |  |  | GTAGAGTCATAAAGAAGTAAACAGTAGGCTTGTATG |
| *lhx1*-CNS14 | 297 | TCCACTCTACCCCACCTCTATATCTAACACTGGTAGACAGTAGACATAACAATGCACAGAAAAAGCCAAGGAGAGACTATTTATATAAAACCCTACACAATAGGGCTATTAAATAAAGTAATAATAAGGAGAAGTAAAGCTGGGGGGGGGGGGAGATTTTCAGAAAGCATCTTTATAAATTTTTATGTAGGAGAGCGGCTGGGGATGGGGATTATGTATGCAGCAAACCCGGTTCAAAACCCGGTCCAGGCCCTTTTGTATTTTTACCACTGTACAGAACAGTGTGGATAGGCCTGC | CCACTCTACCCCACCTCT |
|  |  |  | GCAGGCCTATCCACACTG |
| *lhx1*-CNS15 | 496 | GGAATATACAGAGAGCTGTAGTTGGAGTATACAGAGAGCTGTAGTTGGAGTATACAGAGAACTGTAGTTGGAGTATACAGAGAGCTGTAGTTGGAGTATACAGAGAGCTGTAGTTGGAGTATACAGAGAGCTGTAGTTGGAGTATACAGAGAGAGATGTGGGAAATAGGGCAAAGTCTGAATTCAGTAACATTCTTGGGCTTTGCTTTAGTAAGACTACATATTGCACAGTATTTCTACGGTTACATTbTACTTGACTAGCCATATTGTTATTTTGAAACATTCCAGCTGCCCTGTTAACATACCCTGTGGAGGCTACACACTGGCAAAAGTAGGTGTACTGtatatatatatatatatatatatatatatatatatatatatGAGAGAGATTACCGTACAGTTGCCCCTATGAGTGTATTATGGAACCAACATAATGTTATTAGCTGCAGTATATACACATTAACTCCTCATTATACTGCTAGCTTTGGGGGTGG | GGAATATACAGAGAGCTGTA |
|  |  |  | CCACCCCCAAAGCTAGCA |
| *lhx1*-CNS16 | 546 | ATTACAAGCCCAGAGTGCAATACCTAACCTTGAATAAGTGTTTTCTGGTTGTCATCTAAAGATCTAAAGGAAATCCAGTAGAGAATACATGGAGGGGACATACATTATAAGCCATAGTTTTTTATCTTACACGTGTGACTATGCCTATCAAGAACTGCAGGCTCTTATTCTCACTGGTGGGACACAGCTATCTCCCACTGCTGCATATATCAAAGCAAAGAATTGTGTACCTTTTTTGCATTAATATAAATAGCTGTTTAGGATTGATTCCTGGGCTTGATAGTCTATTCTGGACCGTTCGTCATCGCCCATATTTTATATGGATTGCAAATCTAGCTTATTTAAAGCCTGAATATGGCAGCTTTACATATTAAAGGTGCATTTAGAAAGGGCATTTTGTATTCTAATGTTGCAGCAAAGTGCTCTTTGGGGCATTCGGAGAATGATGTCCATCTGATTTCCATTTCCTTTGGTGAAGAAGAGCCGTATATGCATATGAGAATGTAAATAATAACGATGGATGTGGGCACCCTTAAAGATCAATTT | ATTACAAGCCCAGAGTGCAATACCTAACC |
|  |  |  | AAATTGATCTTTAAGGGTGCCCACATCC |
| *lhx1*-CNS17 | 164 | AGATTCCTCAATTTCACACATGCTTACTCCTGAGTGAGTTTCAAATAAAAGGATTAAGGCAAGAGGAAGTGCAGAGGAAATGGGATGTTAGACACCTTGTTAAACGTGCTGTCAGAGTTTGTGGAGCGTTCATTCTAACTCTATTCCACTCTACATAGCGGACA | AGATTCCTCAATTTCACACATGCTTACTCCTG |
|  |  |  | TGTCCGCTATGTAGAGTGGAATAGAGTTAG |
| *lhx1*-CNS18 | 386 | TTAATCTGTGAAGAATTGTGTTGGAGGATAGTAAAAGCTGTTTAAAAAGGTTGCTCAATATTTCATTTTTAGCAATAAATGATAATATTTATGTCTGTAGACTTTTCCCCCATCTCCACAATGAAGAAAAATAAGACTGCTGGAAATAGTCATCAGAAAGGTTATGCTAGGACTTGTCATTTTTTATATCACTGAGCATTCTATTTGACAAGTGATGTCCTCTTAGCTGCTGGGATAAGCCAAGCACCTTTAGTACTGGCAGTAAAAATGACTATAAAAAATACCATATAAGAAAGGGTAATGATGAAATTTAACTGCTCCCTATCACACACCTGGGGTCTGGTAATCTGAAAATAGGGGTCCAGGTGCTTCATCTTTTATTGCCATGTCCTTGTCTGGAAGTTTAAGGTCCTTCCACTGAACCATAACACAC | TTAATCTGTGAAGAATTGTGTTGGAGGATAGTAAAAGC |
|  |  |  | GTGTGTTATGGTTCAGTGGAAGGACCTTA |
| *lhx1*-CNS19 | 221 | CCCTAGAATGCCAATCACAGAAAGGCAGAGCTATGTTAAATATTCTTTTAGATTAATTAGTATTACTGCGAGAAGCAAAAACCTAAAATAGAGTCGCTGCCTCTGATTGAATTAGTAATTGTAAAACAAGCAGATTTATTACATGCCTAGAATATTCTCCTACTGTAGATATTATTTTGCAATGAAATACTTTTTTCTCAGTGTATGCAGAATGGCTAAAC | CCTAGAATGCCAATCACAG |
|  |  |  | GTTTAGCCATTCTGCATAC |
| *lhx1*-CNS20 | 197 | TCTTTCTTATTCTTTGACCTCCTAGGGTCTAATGCCTCTTTAATACTCACACAGGGAAGACAGCGATAGACAATTAATTGCAATGACAATGATAAAGGAAAAGTCACAAAGAGCTATTTTTCCTTCATGTATCACTGTGCATCATTTTAAGATAAATGAAAAGTAATTTTGGCCTTCATTCAAACTAATTTTTCAGGAATGGTGTCAACCTGCTAAAGTAGAGGACCATAGATTTGAGGGGTAGA | TCTTTCTTATTCTTTGACCTCCTAGGGTCTAATG |
|  |  |  | TCTACCCCTCAAATCTATGGTCCTCTACTTTAG |
| *lhx1*-CNS21 | 219 | AAAGCAAAGACCTGCACATACAAATGAATTTGATATTCAATCAATGAAGATTAACTTGCCCAAGGTAAGATGGTTTTTACATTCATAAGGAAGTCAAATACGATTATGCATATGAGGAAATTATACAGAGATGAGTAGAGTATAATATATGATAATTATTTGAAAGGATATACTTACACAGTGATTAGCATATCTGGAGGACCAGGCAGAGATAGAAGA | AAAGCAAAGACCTGCACATACAAATGAATTTGATATTC |
|  |  |  | TCTTCTATCTCTGCCTGGTCCTCCAG |
| *lhx1*-CNS22 | 343 | CAGGGCTGCAAATTCATCAACTCCAAGCGATTTTCTCAATTCTGACAGCTAACTTCAATGTTACGACAAATCTGGCCCCAGAGGATATTAAGCAAAGACACATGAGCCTATAATTTTCTTCTTCCACATGTGAGATGCTATAATGATCAAAAGAAAATGACTTTCTAACTACCACATCTCTTTGTGCTGTTTTCTTCATGCCTTCTGCACTAATTGAATCCCCCCAGAGTCACTGTATCAATTCTAACTTATGTATTAGGATTTAATCAGGCATGTTGAACGACCAGCTTGGAGAGGTGAGGGGTCTTTTGTGAGGTGTCCATCTGCCTCAGACATTAGAGCC | CAGGGCTGCAAATTCATCAACTCCAA |
|  |  |  | GCTCTAATGTCTGAGGCAGATGGACA |
| *lhx1*-CNS23 | 267 | GAAGGCAGAAGGTGACAATAGGGTCATCATGCCTCATTCTCTGAATGGAAGCCTCTCAAGTGAGGCACCATATGCCGCCTCCTGCTTCCCCATATATGGAGTTTAACGGGTGCTGGATACAGAAATTGGCACAATTCATCTATGCAACTTCTCAGCTGGTCTCCACACCCCACTTGACTTCAAAACGACCTTTCCTTTGTGGACTTCTACATAGAAAAAAACTTCTGACATTGCATAATAAATTGTAGCCTGTTCTACATCGGATGC | GAAGGCAGAAGGTGACAAT |
|  |  |  | GCATCCGATGTAGAACAGG |
| *lhx1*-CNS24 | 287 | GTGTCAAGAGAGATGCACACAAAGCAGAAAATAGTATAGGCCTAATTAGAACAATAGAAAAATGTTCTAATATTAACCTTGACCTTGAAACTCtttaaatatttaataatatttGACAAGTTAGCAGTATAGTTCCCTTTGCGTTCTAGTTGAATAAGCAATGTCAGTAGTGAAATCTTTATTTCTAACATGTAAAAGTAAAAAAACAATATGCAATATATTGTATAAGTATTCGCTATGTGATCCTAAATTATAGCAAATACTCAAGTGATTCAGTGCATCCCTAG | GTGTCAAGAGAGATGCACA |
|  |  |  | TAGGGATGCACTGAATCA |
| *lhx1*-CNS25 | 424 | GGATAATGGGAAGAGATGGTTAATGGGAACAGATCAATGTGATGTGTTGGTACCATACCAAATAGGCTTTTAATTTTCTGGGGAGGTTTGGGGCGTCTCTGCAATGCCTGCATCAATCATCATCATGAGAGCTTCAGGTCCTAGCTCCCCAGGGTCCTTTTTTTCTAAAGGGCTCCTTTTCATTTTCTGACTGGGGGCTGTTTTACCTCCACCACGTGTCATGCAGACTAATTGCTGCCTTGTTCCTCTTATGATGATAAAACACATGTCATATCCACACAGAGCTTTACCACAATAATTTTAATAGGCAATGTAGGAAACATACACATTTTATCTTTGGCTGATATAAAAAATGCACACAGCTTTTGCTGACTTTTGAAACAATACATTTGTGAAATAAATAGCATAATACAAGAATGTGTGG | GGATAATGGGAAGAGATGG |
|  |  |  | CCACACATTCTTGTATTATG |
| *lhx1*-CNS26 | 361 | TCTGTACATCCCACTGCTTCCCTATACATAAAGAGATCTCCAAAGACTTTCTTGCATTGACCTTGAATACCAGTGTGCATAGATATCATGTTCTTTAGTAATCCTCTCTTTTCAGTTGAAAATAAGCACATTGTGTTTATATTTTCCCATATGATATATACTACTGATTTTTGGTTATTTTATTACTTTCACATTTGAGAGAATATTACTTGTGTGCGATTTTTTAGGAAACTTATTGCTGTCTTCTGGCCTCTAGTCAAAATAAACTAATTTTAAATAGTACATATTACTCTGTCCACTCTTTTGCCACAATTTTGACACAATTTTTTTCCCAGCATGCTGAGCCGTAGTGGTAGTGTCC | TCTGTACATCCCACTGCTT |
|  |  |  | GGACACTACCACTACGGC |
| *lhx1*-CNS27 | 442 | ACCAGTCAACCAGGAACTGCAACTAATAATCGTAAGATCTATGAGCTGTAGAGAGATATATTAAAAATTGTTAACTGGATTGGACAATAACCTTTTTATTACAAAATATGGATTTCCAAACTTGAGGGGTCATTTACTTTGTGGGGTAGGCACATTCTCGCATTAATAGGCACACACAGGAATCAATATCATCAGGAAAAATACAAAGTACCGAAAGGTAGCCCACACAGACATGGAAAGAGCATTCAAACTCCTTGCAGGTACTGTCCAGTTCAGAATCTATCaggcccaaactgctctggccagcccaataagtagtatctgtctatggcagggacccccaaccttttttactcgtgggccacactgaaatgacgcaaatgttggTTATTATCAAATATTAAAAACCTTTTCTGAAAGTTAGGTATCCTATAGCCCAGAG | CCAGTCAACCAGGAACTG |
|  |  |  | CTCTGGGCTATAGGATACC |
| *lhx1-*CNS28 | 443 | GCCTGGACTGCGGGATCATAGTGTTCTGTTCCTTTTATTTTAATCATTTAGATACTGATATCTAGATATATTAGATATAGATAACAGTTGATGGTTTTAACATAGTTCGTGGAGACATACACTACAATAAAAAAAACTGGTATTAATTTGTGATAAAGTCAATTAAATATGTGAATAAAACGATTTATTTAAAATTCAATCTCATAAGGTGTTGCTTATTTCTATTAATATTTCTTTGTAAAAATGTGCATGTGTGTGTCTGTCCCTTAAGTCACCAGTCATGAGTTACATTATTTATGTAATGTAATAAAATAAGCCTTTCATTCTACATAAGGTCTTATGCATTTCATTTTTTTTTGTACAGTTATTCACATATTTACTATAGTCAGGTTTACCAGGATCCATTTATTAACCTTCCTGTATGCTTTTGGAGTATGTGAGGA | GCCTGGACTGCGGGATCA |
|  |  |  | TCCTCACATACTCCAAAAGC |
| *lhx1*-CNS29 | 259 | ATGCATTACATTTGTTGCTGACTTAATACAATATGCATATTTGTACTTTCCAAGTAGCTGGCAACCCTTAATCAATGTTAATTGGCTTTAATTAGCATATGTTAATTAATGATATTTACTACAAATTAAAGGTATGAGTGCTCCCAGAGGGTTTCCTGTTCATCTAATTAGTGCCAATTAGCTGCAATTAACACGTGTAAATTAGATGCCATTGAACCCAAAACAAACAGCTTACAGTCCGTTGTATTCACTTGCACTT | ATGCATTACATTTGTTGCTGACTTAATACAATATGC |
|  |  |  | AAGTGCAAGTGAATACAACGGACTGTAAG |
| *lhx1*-CNS30 | 177 | AAATAAATAAATATTCATTTTATAATCCTGACAAATCTATGTATGGTAGAAAATCTGTGAATATTATGATTATTTATAGAAATAAAAAGAGAAAGCCATATTTGATTCCATTATATGATGTAAATATTAATGGTACTTAAATACAAGCACAAGTAATTGCATTCTGGTTTTGGAAAA | Non-Amplicon |
|  |  |  | Non-Amplicon |
| *lhx1*-CNS31 | 431 | CCCCAGTTTGTCCTTAGTCTAGCCCTCCCCAACCTCAGGGAAGCAGAAATCCATTTTACATTCAGATGGTTTCCAACAATATCTGTTGTAAGAGAGCAGCTGAGATGTAGCTTTGGGCACAAATTTTGGAGAAGCAAATATGTAAATTTACTATGTTCTTTATTATGTCTTAAAACATTTGTGAATGCTTTACTTTATTTATTATTACATTATGCTTTCTGTATTAGataattatattcaaaaatcaataaacatttattaaattaaaaaaaaagaaaaaagtaattttaGTGGGTTTTTTTCATGATTTTCCCAAATTGCAAAATTCCAACTTTAGATTAAATCTTCTCCTAAATCTCTGAACAAGCATATATCAAAATTGGCAACTATTAGCGTTAATATACCTTCTGCCACACTTCTAAGCATGGCCC | CCCCAGTTTGTCCTTAGTC |
|  |  |  | GGGCCATGCTTAGAAGTG |
| *lhx1*-CNE32 | 383 | CATGTTAAGTCCGTGTGATTTTCTTGTGGCATCAGAAATTTATTTGCAAGACCAAGTATTTTAAGTAAAACCCTTTTTTCCCTGCTGAGTTGACCATGTTTTTGAATTTATGAAGAAAGATGTTTTAGAAGAAACTCCCAAGGGTCTCTCATCAGTTGTAAATCTCAGCTCTTTTCCCCCTGCAACACTTCATGACACAAAGCATGCCAAAACAGCTTCCTCTTGCCTATGCATTAGCAATCTATTTTACCTTTCTGACATCTCTCACACATAAAACCGTAAGAGAGAAATAAAATATACGTCTCTTGGTGAAAATAAAGCTTTGCCTCCCTCCTGATCTATAGGATATAAAATAAAGCCCTGGCTTGCAAGTGGCTTTTCCTTATTCTGTGGTTGCCTTTGCACGTAGGGATACAATGAAGATGGATTAGAGCAATGGG | CATGTTAAGTCCGTGTGATTTTCTTGTGG |
|  |  |  | CCCATTGCTCTAATCCATCTTCATTGTATCC |
| *lhx1*-CNS33 | 1228 | GTGAGGGGTTAATGGAAATATAAGAATGACAATGAGGTGGGATTATTAAAAGGTTTATAAACCCGCAGAGCTGGGGGTAATGGAGGGTTAAAAGATCATTAAGACGAGGTTAGATTGGAACAGCCTCAGTGTGAGGGTAATTAGGAGTAATCTTGTCAGGGTAATAAGAATTAACTCAATCATTCTTGACCCAAAGCTAGAAAGACAAGCAAGAATGAAGCTAAATGCCTCTAAATGTTTCACTCACTACTAACTGAGAGGGCCGCTAGCCTGTCTATAATATAAAATCTATTTTGTTTATGTTTTCATAGAGAATAACACATTTCTGCACATTTGTGCTCACTGGAAATATCCCACAACGACCCAAAGGTTTAGAACGGTTATACCCCCTCCCCCACCCGCGTGGGGGAGCAGTCCTTTAGCCTGCAGCTAAAGCAAAAGTTAATATGTGATACTAGGGGCCATGTAGATTGATTGTATATAGTCTACTCATCCACTAATTAAAAAACGTTGTCCTGTTCATTTTTACCAGTATTTATTTTTCCAAATATAACTTTGCGTCATAATATTTTTCTGCTGTGACGTAATATACAATGATTTTCCGTTAGATAGTTTCTTGCACAGCACTTGGGACTTAAATCCATTCGCCTGAAATTCTAACATCTTATTTCAGTCAGAATTATGTGTATTTCATCTCTCGCTCAGCATTGCATTTCGCCTATTAGATTGAATGTCATCTTCAGCAGTTGATGTATGAAAATTAATATCGAGACAGTTTAAATGTGTACCATGTAGCCATTTAAATATTAACCTTAAAAAAAGAAAACGTTAAAAACGTGATATTATACTCAAACGAAATACTTTTAGATGCTCCACTGCGGTTTCCGTTACAATGAGACTGGATTTACTTATCTATATTTGCAACTTTGTAAGGAACTCGTTTTTAACAACAAAGATAAACTACAATTTAGAGCAAAACACATAAAACGCAAATTTATGTTTGGTTTTAAATTGGAATTATAAAGACAGTACAATTTTCTATTTTATAATAACCTACTTCAAGCAAGTTTATAAACTTTTCAAATTCTGTTTCTGTATATACAGTATATTTATCGATTAATAGCTCGGAGAGCAGAAATTAAAAACATAAAAACGAAAACCAAACTATAAAAATTAATACTGCAAATCAATGGATAGAGAAGCAAACATATCTACTAAACAGGAGCGC | GTGAGGGGTTAATGGAAATA |
|  |  |  | GCGCTCCTGTTTAGTAGAT |
| *lhx1*-CNS34 | 258 | GTGACTTTACTAGGTTTGGCTGTCCTTAGATTCCTGAGATCGTAAGTTCAGCTTGATGATTTACTGCAAAATTGCTTCTAAATTGAAGTAGAAAATGTTTTGCAGACCTGGCAGCTTTCAAGCTTTGCACCCATTTGTCACCCAGCATTTCCCTTTGTGTATGGAACAAGAATTAATTAGGCTGCTCGGCAGGAAAACTTTATGGCTATAATTTATATACTAGGAATAACCGTTAAAGAGACATCTATGCTTTGTT | GTGACTTTACTAGGTTTGGCTGTCCTTAGATT |
|  |  |  | AACAAAGCATAGATGTCTCTTTAACGGTTATTCCTA |
| *lhx1-*CNS35 | 580 | AATTCTTTTCCTGACGAATTTTGGGGTCTGAATATTATGGCTTATTGTTATTCTGAGTTTATAATCTCTGCCGTGCTTTAATAATTTTCCCTCATGCCTGGGTAGTCCGCGAGTTGCACAATAATCACTCTCACAGCTTTGTAATCAATGCCCACAGTAATAGGATTCCTCTGGCCACTGGCAATTAATAAATTTGTGTCTTTTTTTAAAACACTAGCAAGTCGGGTCTGAAATACGACTGACTGGCTTTGCTCATTTGCATATTTATTGCAGTGGAAAAATTCTTGCCGAATGATTGATGTCAGACCTGCCTCTTGAATTCCAAGCAGCACATTATTATGAAATGAAAAATGCCGGCCCTAGAGTTGATTAAAGTTGAAGACAGTGGAATCTGTGTTTTTTAAGATTGTGGGAGAATTAAAGGCATATTTTAATGGATGTTGACAAGAAGTGAGCCAACAAAAGCAAAGCAAAGGATTTGCTTGAAAAGATTTAATCAGACGTGTTTCTTGGGGGATTAATTGCTGGGGATGACTGGTGCAAGTGGCAGGCTTTGTGGATTTAAATGAGAATTGTTCTT | AATTCTTTTCCTGACGAATTTTGGGGTCTGAATAT |
|  |  |  | AAGAACAATTCTCATTTAAATCCACAAAGCCTG |
| *lhx1*-CNS36 | 383 | TGAGGGTAACTGCCATTGTGTGTTTTTTTTTTAAATAACCTTTAGAATTTATGGCTATCTTTCTTTCAAAAAGAAACCAAATGTTAATGGATATACATATTCATTATCCCAGTAAAAAAAAAAAAATCCTGACTGCTGTATTTAGATTACAGTGAGACAATCAGATGAAGATGTGACCTTTACCAAACCCTTGCCATTTTCTCAATCTCTCCATTCATAGTGACCAGCAAAATTACTAGAGCAATTATAAAACATTTGCATTGCACATCAAACTTTTTCGTTATGTAATATTTAAAAATCCAGCAATACACACTGAACTGGATGATTATATTGTCCATTTACCAGTGTTGTTCTTGAGCACTGTTATTGGTCGAAGGGGTGCC | TGAGGGTAACTGCCATTGT |
|  |  |  | GGCACCCCTTCGACCAAT |
| *lhx1*-CNS37 | 988 | AAGATAGCACCAGATGACCAGCCTTTTATGCCGCTTCATAATTTTAACTCTGATAAATTCCAAATTCAGAGTTATGAGTAGTAGCATCTTATCTTTTTTCCCCCCTGACTGGTTAATTGGGGTGGCGGGGTTAATCTAAATTAAAACTTAAACTAATAGAGGTCAGTAAGGCAACAGAAACACCTGAGTAAAACAAATGAGGCTGCCAGCCGCAACTTTATCTTGGCCGGCTCCCAGGAGGCGATTGGCAGAGAATTACAGTCGCTCTGGCATTCTGCAAAGGTGAATGATGGGCGACACTTGTCAGTGCGCAGCATCTCTACAAATGTTCCAGATTGCGGGACTTCCGTGCCAACTGCTGCCATCCGTGTCGAGTGAATGGGAGCAGGAGCAGTTGTCAACATCTGGCATCACAGCAAAGAATGGGTGCAAGGCTGGGAACCCTGCAGACTCTAAATATGGTGCTGGGAGCTGTGGAGGCTTTAATCACTTACCGCTCCTTGGCAGCAGGCTACAACAGCATATCCCAGCCTCGTTCATCACATGCAGACATCTGCTGAGCCGTAAGGCAGCTACACATTCATTTTCTTTCCTTGACCCCCCCCCCCCCATTATTTTTCTCTTTTTCTTTTTCATACACCTTCCACCACTCCTCCCTCTATTCCAGTCTCCCACCAAAACTGTTTATTGGGAGACATGATCTTTAAAGATTACCACTTCAGTGTGTGCTAGTAATTGTAGGGAAGAGGGGCCCCCTGAGAGACACCGCATCACACCTGTTCAGATGACCGGGTTAATTTCTTCTGTTGAGTGGGATTGGGCACAAATTCAAGTTGATGCCAATTCAACAATAAGGATAAAGAAAATTGCTGTTATGTTTTCTGTCATCTTTTACATTAAGCTGAATGTTTTTTTTCTCATTTGTCAGTAGAGCAAGCCTGTGTCGAGTGCCACACTTAGGTGCTGTCAAATGCACAGCAAAGGGTTT | AAGATAGCACCAGATGACCAGCCTTTTAT |
|  |  |  | AAACCCTTTGCTGTGCATTTGACAG |
| *pax2*-CNS | 793 | TGTGTATTGTGAGAGCTTTGGAGTAGATTTCTACCTCAACCTTTTCTACCCCCTAAATCTTATCTTTAAGGTTGTTTGTCTTAATAAAATATTAAATTAGAGAAAAATTCTTTTGAAACTGCCCAAAACTATAAAAGATGAAATGATGAGAAAGCATGCTTGAGATTGGAATTTCTCTTCTCCCAGAGTCCCAAAGTTTATTACAATGGATAACCTTTATTTACTTTCATTAGACTATTAAACGCCAGCACAGTCATTTTTTCCCCCTGAAACTTAAAGCAGGGCCCATAAAGCAAATCTAAAGCCTAATTGAGTTCATTTTAATTTCTCTCCGATCACAGCGAATTACTCCGGTGATAAATCAGGGGGCAGCTTCACCCCCAGTAGAAGGCCTAATTTGCAGCTAATTACAAAACTGATTTCAACAGGAAGATCAATACGAAAAGGAATTGGAAATGACTAGGCTATAGCGTCCAGGCTTAGAGGTGGGGGGCGTGGATGGGAAAAATGGGCTAGAAGAGCAGAAAATCAGTTTTAATTTAATGTAATATTAATCAGGCAGAGTTTTCTCCCCTTTTCTTCTCGGTTTGCCCTGTTTATTATCCCTTTGAAAAGGCAGCTGCAGAGACCAGGGTCTGCTTTAGTGAAAACCATAATAAAAAGGCAATTACGAGATTGTTTCGGACAGGACACCCTTTCAGATGCAATGACTCTTTCCCTCCCACCCTTCTGTTAACCATGTGCTGTGGCCCACTCCCCAATTTCATACCCGACTGCACAGGGAAAGGGGAAA | TGTGTATTGTGAGAGCTTTGGAG |
|  |  |  | GACGTGTCCCTTTCCCCTTT |
